# Revision of the NIST Standard for ^223^Ra: New Measurements and Review of 2008 Data

**DOI:** 10.6028/jres.120.004

**Published:** 2015-03-11

**Authors:** B. E. Zimmerman, D. E. Bergeron, J. T. Cessna, R. Fitzgerald, L. Pibida

**Affiliations:** National Institute of Standards and Technology, Gaithersburg, MD 20899

**Keywords:** anticoincidence counting, ionization chambers, liquid scintillation spectrometry, radium-223, standards, traceability

## Abstract

After discovering a discrepancy in the transfer standard currently being disseminated by the National Institute of Standards and Technology (NIST), we have performed a new primary standardization of the alpha-emitter ^223^Ra using Live-timed Anticoincidence Counting (LTAC) and the Triple-to-Double Coincidence Ratio Method (TDCR). Additional confirmatory measurements were made with the CIEMAT-NIST efficiency tracing method (CNET) of liquid scintillation counting, integral γ-ray counting using a NaI(Tl) well counter, and several High Purity Germanium (HPGe) detectors in an attempt to understand the origin of the discrepancy and to provide a correction. The results indicate that a −9.5 % difference exists between activity values obtained using the former transfer standard relative to the new primary standardization. During one of the experiments, a 2 % difference in activity was observed between dilutions of the ^223^Ra master solution prepared using the composition used in the original standardization and those prepared using 1 mol·L^−1^ HCl. This effect appeared to be dependent on the number of dilutions or the total dilution factor to the master solution, but the magnitude was not reproducible. A new calibration factor (“K-value”) has been determined for the NIST Secondary Standard Ionization Chamber (IC “A”), thereby correcting the discrepancy between the primary and secondary standards.

## 1. Introduction

In 2010, the National Institute of Standards and Technology (NIST) published the results of the first-ever standardization of the α-emitting radionuclide ^223^Ra in secular equilibrium with its decay daughters that had been carried out between 2006 and 2008 [1]. In that set of studies, which we refer to in this paper as the “2008 standardization”, a calibration factor for NIST Ionization Chamber “A” (IC “A”) was determined from the measured response of the chamber for ^223^Ra solutions in the standard NIST 5-mL ampoule geometry and the calibrated activity of gravimetrically-related solutions as measured using liquid scintillation (LS) counting and alpha spectrometry with 2π proportional counters.

A companion paper describing the development of calibration factors for several re-entrant ionization chambers (often called “dose calibrators”) using different geometries was also published at the same time [2]. The activity values used to derive those calibration factors were obtained using IC “A” and the calibration factor derived in the aforementioned 2008 primary standardization experiments. Since that time, these calibration factors have been used in clinics and manufacturing sites to assay ^223^Ra solutions worldwide, as the use of ^223^Ra as a radiotherapeutic agent against bone metastases continues to increase [3].

In the summer of 2013, we were made aware of studies being carried out by the National Physical Laboratory (NPL), the national metrology institute of the United Kingdom, in which an approximately +10 % difference was found between their activities obtained using several primary methods and those obtained with the calibration factors published by Bergeron, *et al.* [2] using the appropriate instruments maintained in their laboratory. An aliquot of the same solution standardized by the NPL was sent to NIST and we confirmed that the activity concentration value obtained by LS based primary methods indeed differed by about 10 % from the values obtained using our secondary standards developed in 2008.

This result prompted a thorough evaluation of the data collection and analysis done in the experiments that comprised the 2008 standardization. This exercise did not reveal any errors in the way that the data were collected and treated. Indeed, all the dilutions in our laboratory are done in such a way so as to provide an internal check by providing masses of radioactive solution and diluent dispensed from the pycnometer as well as the masses contained in the respective dilution vessels. For those experiments, the maximum difference between dilution factors calculated from the contained and dispensed masses was less than 0.2 %, with the majority being at least an order of magnitude lower.

Because there were no empirical data to suggest that the ^223^Ra solutions were unstable with respect to dilutions or transfers and because the contained and dispensed gravimetric dilution factors demonstrated such good agreement, no systematic monitoring of the dilution factors by radiometric means was pursued. In some cases, though, ionization chamber measurements were available for ampoules containing master solutions and first-step dilutions (typically a factor of about 5) and the agreement in those cases (viewed retrospectively) was better than 0.7 %.

It was noted during the 2013 experiments done on the NPL solution that a fundamental difference between the original 2008 NIST standardization and the one being done at NPL was the fact that the NIST standard was based upon measurements done on the drug product as it came from the manufacturer (citrate solution), while the NPL standard was based on measurements in which the drug product was initially diluted using 1 mol·L^−1^ HCl [4]. While no direct evidence for solution instability was present in the previous standardization experiments, given the absence of calculational or transcription errors this was hypothesized to be the cause of the observed discrepancy. The experiments described in this paper were conducted in order to attempt to understand the origin of this observed difference between the NIST ^223^Ra primary and secondary standards and to provide a new standardization based on measurements with both massic and radiometric verifications of all dilution factors.

## 2. Materials and Methods

### 2.1 Overview

This study consisted of three separate experiments, performed over the course of about 6 months. The first experiment (Experiment 1) was preliminary in nature and consisted of measuring the same solution that was used in the standardization of ^223^Ra at NPL by LS counting, and in IC “A”, and a Vinten 671 secondary standard ionization chamber (VIC)^1^. The purpose was to confirm the presence and approximate magnitude of the discrepancy between the NIST and NPL values using the methods applied in the 2008 standardization. The experiment was performed solely for indication and was only meant to determine whether or not the suspected effect was present. Therefore, the details are not discussed in this paper. It suffices to say, however, that the discrepancy was confirmed with an observed difference of about 10 % between the LS counting results and the ionization chamber measurements.

The second experiment (referred to hereafter as “Experiment 2”) served as the new primary standardization and also attempted to determine whether possible solution instability effects with respect to transfer or dilution in the citrate solution could have contributed to the observed discrepancy between the 2008 NIST standard and the LS counting results of Experiment 1.

The third experiment (“Experiment 3”) was performed after it was determined that the citrate solution currently being distributed by the manufacturer, and which was used in the first of these experiments, differed from the 2008 NIST solution in that the original solution contained trace amounts of Sr^+2^. The Sr^+2^ was used by the manufacturer in the original solution, but was removed subsequent to the development of the 2008 NIST standard.

### 2.2 Source Preparation

#### 2.2.1 Experiment 2

**Fig. 1:**
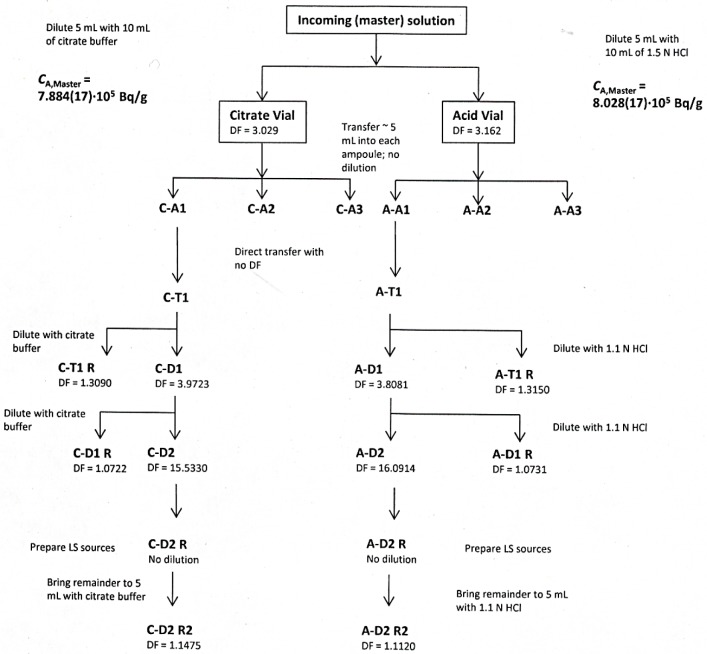
Dilution and source preparation scheme for Experiment 2. The values given for *C_A,_*_master_ are based on the respective LTAC-derived activities for the acid and citrate series solutions, multiplied by the appropriate gravimetric dilution factors. Gravimetric dilution (DF) factors are given.

The experimental scheme for Experiment 2 is shown in Fig. 1. A 10 mL stock solution containing nominally 57 MBq of ^223^RaCl_2_ and between 7 mg to 9 mg each of NaCl and Na_3_C_6_H_5_O_7_ per gram of solution was received from the Isotope Laboratories, Institute for Energy Technology (Kjeller, Norway). An additional amount of the “cold” solution containing no ^223^Ra (or Sr^+2^) was provided by Algeta, ASA (Oslo, Norway).

Two 20 mL dose vials were prepared by gravimetrically dispensing 10 mL of the citrate carrier solution into one of the vials and 10 mL of nominally 1.5 mol·L^−1^ HCl into the other. Approximately 5 mL of the stock solution were gravimetrically transferred into each vial to give 15 mL total. These vials were designated as the “citrate” and “acid” master solutions.

From each of the two master solutions, three 5 mL NIST standard ampoules were prepared by gravimetrically transferring 5 mL of the solution from the respective vial into each ampoule. These ampoules were designated as A1, A2, and A3 for the acid series and C1, C2, and C3 for the citrate.

In order to test for possible losses due to adsorption, the contents of A1 and C1 were gravimetrically transferred without dilution into two new ampoules, designated A-T1 and C-T1. Two additional serial gravimetric dilutions were performed on each ampoule, by factors of about 4 and 16, respectively, to give four more ampoules with designations A-D1, A-D2, C-D1, and C-D2. In each case, the dilutions were done with either the citrate solution (for the C series) or 1 mol·L^−1^ HCl (for the A series).

In order to keep the volumes consistent, the remainder solutions from A-D1 and C-D1, which were used to prepare A-D2 and C-D2, were back-filled with the appropriate diluent (citrate or acid) to bring the total volume to 5 mL. These ampoules were designated A-D1 R and C-D1 R.

The solutions from ampoules A-D2 and C-D2 were then used to prepare liquid scintillation counting sources for live-timed anticoincidence counting (LTAC) [5], the Triple-to-Double Coincidence Ratio (TDCR) method [6], and the CIEMAT-NIST (CNET) efficiency tracing method [7]. For CNET, LS sources were prepared by adding 10 mL of Ultima Gold AB (UGAB, Perkin Elmer, Waltham, MA) into each of twelve 22 mL glass LS vials with foil-lined plastic caps. A total of 0.5 mL of citrate solution was added to half of the vials and 0.5 mL of nominally 1 mol·L^−1^ HCl was added to the other half. The amount of quenching was changed by the addition of 0 to 12 drops of a 1:10 dilution (by volume) of nitromethane in ethanol. Nominally 0.06 g of the corresponding X-D2 (where X = A or C for the acid and citrate solutions, respectively) solution was gravimetrically added to each vial in the respective series. Two counting blanks for each series were prepared in a similar manner, but with 0 drops of dilute nitromethane in one and 12 drops in the other.

The TDCR counting sources were prepared in a similar manner, but with only 3 vials each in the citrate and acid series. No nitromethane was added. A single counting blank was prepared for each series with 10 mL of UGAB and 0.5 mL of either the citrate solution or 1 mol·L^−1^ HCl.

For LTAC, the counting sources consisted of two glass hemispheres in each series, each containing nominally 3 mL of UGAB in a 7 mL hemisphere and 0.35 mL of the respective diluent. In order to investigate possible loss effects due to diffusion of the ^219^Rn daughter from the cocktail, an additional series of hemispheres was made from the acid solution that consisted of the same volumes of the constituents given above, but in a 5.5 mL hemisphere. As with the TDCR and CNET sources, nominally 0.06 g of the respective X-D2 solutions were gravimetrically added to each hemisphere prior to being sealed with epoxy. Counting blanks for each of the three hemisphere sets were prepared with compositions similar to the active sources, with the addition of nominally 0.06 g of the appropriate diluent in place of the ^223^Ra solution.

All LS sources were allowed to properly dark adapt prior to counting.

The ampoules containing the remainders of A-D2 and C-D2 were opened four days later and their contents gravimetrically transferred to new ampoules and the solution volumes brought back up to 5 mL by the addition of the appropriate amount of the respective diluent in order to minimize the need for volume corrections when measured by HPGe γ-ray spectrometry. These were designated A-D2 R2 and C-D2 R2. The purpose of this transfer was to check for possible activity losses during the preparation of the LS sources.

#### 2.2.2 Experiment 3

**Fig 2:**
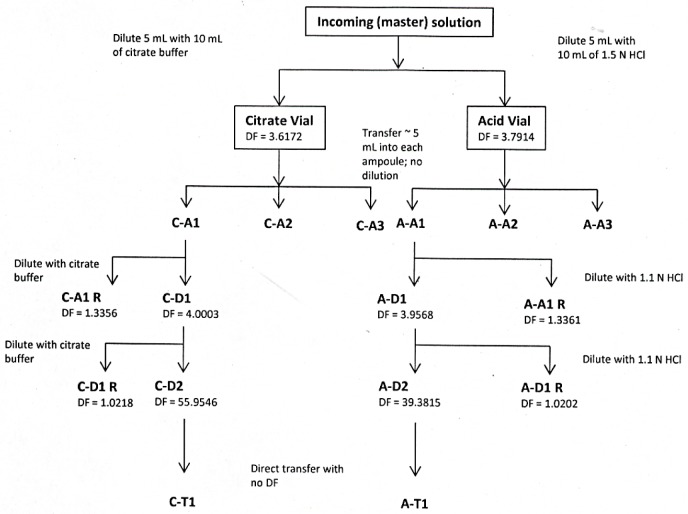
Dilution and source preparation scheme for Experiment 3. Gravimetric dilution factors (DF) are given.

The preparation scheme for Experiment 3, shown in Fig. 2, was very similar to that of Experiment 2, with the primary exception that a diluent that was identical (according to the manufacturer) to that used in the 2008 standardization experiments (i.e., contained trace amounts of Sr^+2^) was used to prepare the “citrate” sources. As a control, a new set of “acid” sources was prepared in parallel, as was done in Experiment 2.

As part of the investigation into possible solution composition effects, an effort was made to have a larger total dilution factor at the final step in order to replicate more closely the conditions used in the 2008 experiments, while maintaining the same level of activity at the respective counting times as the corresponding level of the dilution scheme used in Experiment 2. Therefore, it was necessary to prepare the sources on a much more compact schedule in order to avoid excessive decay time between dilutions.

Another change made in Experiment 3 was that no sources were prepared for primary standardization measurements; instead measurements were made on a comparative basis. Additionally, a simple transfer between ampoules with no dilution was performed as the final step in the scheme in order to test whether or not possible transfer losses could be enhanced by larger dilution factors.

### 2.3 Measurement Methods

#### 2.3.1 Ionization chamber measurements

In order to both monitor the dilution factors during each step of source preparation and to determine new or revised calibration factors for the various secondary standard measurement systems maintained at NIST, measurements were made in NIST IC “A” [8], the NIST automated ionization chamber (AutoIC) [9, 10], and the VIC. Measurements were also made using several commercial re-entrant ionization chambers (“dose calibrators”) and those results will be reported separately.

For the IC “A” measurements, ampoules X-A1, X-T1, X-T1R, X-D1, and X-D1R (again, where X = A for the acid series or C for the citrate series) were measured 40 times each, in four groups of 10 measurements, alternating with 5 groups of 10 measurements of ^226^Ra reference source RRS50 or RRS10. The results were analyzed as a ratio of the response of the ampoule to the response of the RRS. After correction for background, the resulting ratio was used to derive a calibration factor, or K-value, defined as the activity of a given radionuclide that would produce the same response as the RRS.

For the AutoIC relative measurements, ampoules X-A1, X-T1, X-T1R, X-D1, X-D1R, and X-D2 were measured 50 times each, in five groups of 10 measurements, alternating with five groups of 10 measurements of a ^226^Ra reference source RRS50 or RRS10. The ampoules were measured in groups such that the ionization chamber response ratio was measured directly for “C” series vs. “A” series ampoules and dilution factors were measured directly as, for instance, the response ratio of ampoule A-D1R to A-D2. After correction for background and decay, the resulting ratios to the radium reference source were used to derive K-values for the AutoIC, and to determine radiometric dilution factors.

**Fig. 3:**
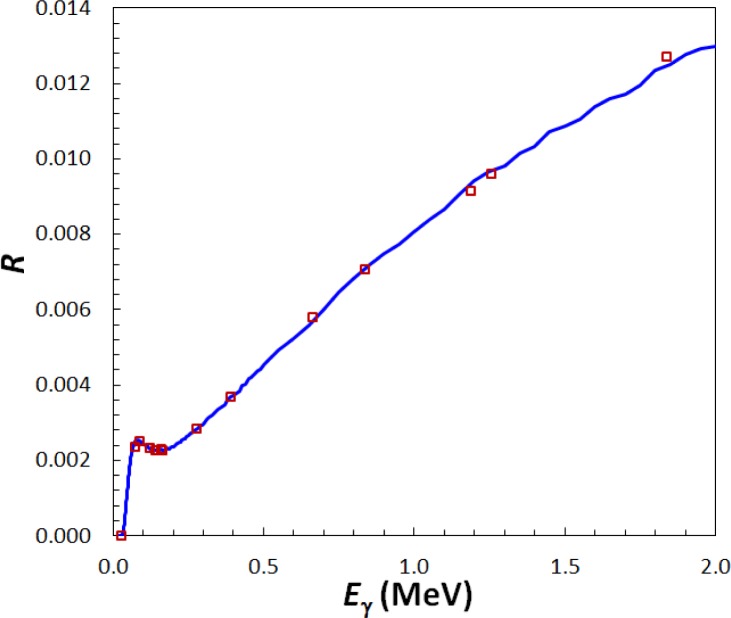
AutoIC response as measured using NIST standards (squares) and calculated using Monte Carlo (line). The uncertainties on each datum lie within the respective symbol.

A theoretical efficiency (K-value) for the AutoIC was calculated to provide an independent value for the ^223^Ra activity. The AutoIC response was determined using a Monte Carlo model fit to previously-measured responses. That is, measured responses were plotted against their effective energies for 14 different radionuclides. These included recent LTAC primary standardizations for ^60^Co, ^57^Co, ^99m^Tc, ^67^Ga and ^177^Lu. They also included K-values transferred from IC “A”, corrected for the changing sample-holder height, of ^125^I, ^201^Tl, ^109^Cd, ^123^I, ^139^Ce, ^203^Hg, ^113^Sn, ^137^Cs, ^54^Mn, ^59^Fe and ^88^Y [10]. The uncertainties on the experimental K-values had a median value of 0.5 %. The Monte Carlo calculations were carried out using the DOSRZnrc user code from EGSnrc [11] in which the exact gas pressure and wall thickness were adjusted to match the model to the data. The experimental and model efficiencies are shown in Fig. 3. For energies above 73 keV, the differences between the data and model had a standard deviation of 0.7 % and a root mean square value of 0.9 %. The ^201^Tl point (effective energy 72.5 keV) was 4.3 % below the model, however Michotte *et al.* [12] have noted that the electron capture probabilities are in question, based on their own similar ionization chamber modeling. The uncertainty in the efficiency, based on the residuals, was taken to be a quadratic function defined by the points (E [keV], u [%]): (72, 1.5); (950, 0.8); (1836, 1.5).

This Monte Carlo model was used to calculate the AutoIC K-value for ^223^Ra in equilibrium with its daughters. A total of 158 γ-ray and x-ray lines were included in the model. The data were taken from the Decay Data Evaluation Project (DDEP) evaluation [13]. About 40 % of the IC response was due to x-rays, 0.8 % due to bremsstrahlung and the rest from γ-rays. As a check of the input data, the model was run a second time using Medical Internal Radiation Dose (MIRD) data from the National Nuclear Data Center (NNDC) [14] instead of DDEP. The MIRD data format is based on the Evaluated Nuclear Structure Data File (ENSDF) data and includes atomic transitions, but is not updated as frequently. The calculated response decreased by 2.3 %. Most of this loss (1.8 %) was due to the absence of the ^211^Bi x-rays in the ENSDF evaluation. This leaves 0.5 % difference between the results from the two data sets. Since the DDEP evaluation is more current and complete, it was used for the final calculation.

Measurements were made in the VIC on six ampoules of the acid solution from Experiment 2 over a period of 27 days. For each measurement, the ampoule was placed in the ampoule holder, and the current was directly read from a Keithley 6517 electrometer using a LabVIEW interface; 100 readings were acquired at 2 s intervals. The currents were corrected for background and decay corrected to the reference time before being averaged. Typical measured currents were in the range from 2.5 pA to 17.5 pA.

For Experiment 3, ampoules X-A1, X-A2, X-A3, X-D1, X-A1 R, X-D2, and X-D1 R were all measured in both the VIC and the AutoIC using the same conditions as for Experiment 2 in order to determine radiometric dilution factors.

#### 2.3.2 Live-timed anticoincidence counting measurements

The NIST LTAC system has been described previously [15]. The ^223^Ra massic activity was determined by live-timed anticoincidence counting (LTAC) of the entire ^223^Ra decay chain, using a method that was previously applied at NIST to the ^229^Th decay chain [16]. The extending dead time of the system was set to τ_E_ = 50.5 μs. For the present case, 3 γ-ray gates were used: G1 at 270 keV (236 keV to 295 keV), G2 at 410 keV (380 keV to 440 keV), and G3 at 800 keV (780 keV to 890 keV). The response in gate G1 corresponded mostly to photons following *α*-decays, G2 corresponded to photons emitted following a mixture of *α*-and ^211^Pb *β*-decays (*E*_β_ = 962 keV), and G3 corresponded to photons emitted only from ^211^Pb *β*-decays (*E*_β_ = 535 keV).

Each of the four (two for each of the two solutions) ^223^Ra 7-mL hemispheres was measured twice. Each of the two 5.5-mL hemispheres (acid solution only) was measured once. Each source measurement consisted of 9 to 26 loops through 16 LS thresholds for a total of about 8 hours of counting data with a total of about 3 × 10^7^ LS counts. The extrapolation was then carried out using 9 of those 16 LS thresholds, with a total of about 2 × 10^7^ LS counts. The net LS count rate over that threshold range, relative to the extrapolation intercept, was 0.929 to 0.993. Background measurements were made in a similar way for counting times between 2 hours and 8 hours. The relative LS background at the lowest threshold used for the fit was 0.8 %. The relative NaI background on the γ-ray singles in gate G2 was 6 %.

#### 2.3.3 TDCR measurements

The TDCR sources were measured in the NIST TDCR system [17] using the MAC3 module to handle the coincidence logic [18]. Several changes have been made to the TDCR system since 2008, including the construction of a new sample chamber, that have resulted in higher overall detection efficiency and lower background.

The detection efficiency was varied using gray filters. The extending dead time was set at 50 μs. Each source was counted three times for 500 s with each filter for a total of 9 data points per cycle. All but one source were counted a second time under the same conditions for an additional 9 data points, and two of the sources were counted a third time for 600 s at each counting point. One of the sources from the acid series was allowed to count continuously overnight in 500 s bins following the day of preparation to investigate possible cocktail stability effects. The total number of triple and double coincidences collected during each measurement was between 8.6 × 10^5^ and 1.1 × 10^6^. Counting dead times were below 20 %.

#### 2.3.4 CIEMAT-NIST efficiency tracing measurements

The sources were serially counted in all three of the commercial LS counting systems maintained by our group: a Beckman Coulter LS6500 (Beckman Coulter, Pasadena, CA), a Packard 2500TR, and a Wallac 1414 Guardian (both Perkin Elmer, Waltham, MA). Each source set was counted for 10 cycles of between 600 s and 900 s before being moved to the next counter. The total number of counts in each spectrum (open window) was more than 10^6.^ The sources were agitated before being inserted into each counter to ensure proper mixing.

Although the CNET technique was applied to these samples, no ^3^H tracing sources were prepared. Instead, efficiencies were calculated based on model calculations with estimated ^3^H efficiencies (see Sec. 3.4.1).

#### 2.3.5 HPGe γ-ray spectrometry measurements

Ampoules X-A1, X-A2, X-A3, X-T1 R, X-D1 R, and X-D2 R2 from Experiment 2 were all counted using four different high purity germanium (HPGe) detectors. Each source was counted in six different geometries, with source-to-detector distances varying between 35 cm to 90 cm, for a total of 8 measurements per source. The counting time for each measurement was approximately twelve hours.

For Experiment 3, Ampoules X-A3, X-D1 R, X-D2, and X-T1 were measured using the same counting protocol as above.

#### 2.3.6 NaI(Tl) well counter measurements

Ampoules X-A2, X-A3, X-D1 R, X-D2, X-D2 R2, and X-T1 R from Experiment 2, as well as blank sample holders (including specially constructed plastic ampoule adapters) were counted at least 3 times each in a Wallac Wizard 2480 (Perkin Elmer, Waltham, MA) gamma well counter over the course of about 2 months. The live counting times were adjusted between 30 s and 1.6 × 10^4^ s in order to ensure at least 10^6^ counts in the spectra. The dead times for the first-level ampoules (e.g., X-A2 and X-A3) were extremely high during the first set of counts and were not used in the analysis.

For Experiment 3, ampoules X-A1, X-A2, X-A3, X-D1 R, X-D2 R, and X-T1 were measured over the course of about two months using the same counting protocol as for Experiment 2.

## 3. Results and Discussion

For all calculations requiring nuclear and atomic input data for any of the members of the ^223^Ra decay chain, with the exception of the γ-ray spectrometry, values were taken from the Decay Data Evaluation Project (DDEP) database [13]. Gamma-ray emission probabilities were taken from recent data provided by NPL [19].

Unless explicitly stated, any uncertainty cited in this paper corresponds to a one standard uncertainty interval. All individual uncertainty components are given as estimated experimental standard deviations (or standard deviations of the mean, if appropriate), or quantities assumed to correspond to standard deviations regardless of the method used to evaluate their magnitude. Individual relative uncertainty components are combined in quadrature to give the relative combined standard uncertainty, *u*_c_, provided with all final activity values.

### 3.1 Review of Data from 2008 Standardization

The analysis of the 2008 standardization data followed a rigorous approach that is taken with all standardization studies and involved many internal consistency checks. Nonetheless, a thorough review of the data from the 2008 standardization experiments was an integral part of this study in order to try to understand the origin of the break in the link between the ionization chamber measurements and the primary standardization measurements. As a first step, the individuals involved in the 2008 experiments re-analyzed the data for which they were responsible. No discrepancies in the recording, transcription, or analysis of the data were found. The data sets were then exchanged and re-analyzed by a person not associated with the analysis of those particular data. Again, the same results were obtained.

The next step was for a member of the NIST Radioactivity Group, who was not involved with any aspect of the 2008 studies, to independently analyze one of the LS data sets and the data from both the IC “A” and Vinten 671 measurements. In both cases, the calibration coefficients obtained during the 2008 experiments for the two ionization chambers were recovered.

This internal consistency led us to the conclusion that the LS-based primary standardization measurements, the alpha spectrometry, and the calibration coefficients calculated from the measured ionization chamber currents and the activity values were valid for those experiments.

The data from Experiment 1, which used a ^223^Ra solution that was prepared at NPL with a 1 mol·L^−1^ HCl diluent showed that the VIC and IC “A” calibration factors both gave the same relative bias relative to the NPL result. The LS measurements, however, were in excellent accord with the value reported by NPL. Since we applied the same methods in both the 2008 standardization and Experiment 1, this result provided confidence that the LS counting done in 2008 was correct.

All of this information led us to the hypothesis that although the gravimetric dilution factors in the 2008 experiments were known to better than 0.1 %, at least one *radiometric* link between the master solution and the serially-diluted solution that was used for the standardization was broken. Stated alternatively, about 10 % of the activity from the ^223^Ra decay chain was lost without a corresponding loss in solution mass. This effect went unnoticed during the 2008 standardization studies because the same (now known to be incorrect) K-value for IC “A” was recovered to within 0.4 % during two independent experiments.

### 3.2 Verification of Dilution Factors

#### 3.2.1 Experiment 2

Since it was postulated that the source of the discrepancy between the original ^223^Ra secondary standard and the new data from NPL was loss of activity during dilutions or transfers due to solution instability, much effort was expended in monitoring the dilution factors in as many ways as possible. The data in Table 1 show the measured activity ratios of sources from the different dilution levels in both the acid (Table 1a) and citrate (Table 1b) series, along with the corresponding gravimetric ratios.

For the acid series, the gravimetric and radiometric dilution factors all agree to within the respective uncertainties. Because of the much lower activities in the diluted solutions (e.g., A-D1, A-D2, A-D2 R), the uncertainties on the measured radiometric dilution factors are much higher than those further up on the dilution chain. For the final dilution step, the uncertainty on the radiometric dilution factor on A-D1 to A-D2 was 0.25 % as measured by the AutoIC. To ensure that no ^223^Ra was lost during the dispensing into the LS vials, the remainder of the solution (A-D2 R) was diluted to bring the total volume to 5 mL and measured again. This time, the radiometric dilution factor, although in excellent agreement with the gravimetric dilution factor, had a much higher standard uncertainty, namely 1.8 %, because it was driven by the large uncertainties (due to low counting rates) on both A-D2 and A-D2 R2. Although this indicates that the 10 % loss effect that was in the 2008 data is not present in the acid solution, the method used allowed the final dilution step to be radiometrically measured only to within 1.8 %.

The situation with the citrate series is somewhat more complicated in that some of the radiometric dilution factors determined by different methods for the same solution are not in agreement within their respective uncertainties, as seen in Table 1b. Moreover, for the dilution from C-D1 to C-D2, the gravimetric and (AutoIC-based) radiometric dilution factors are not in agreement. Agreement exists, however, between the gravimetric dilution factors for the remainders of each of the solutions (C-D1 R and C-D2 R) and the radiometric dilution factors from both γ-ray spectrometry and the NaI well counter. But in this case, the uncertainties on these ratios are high, about 1.8 %, thus the degree of agreement should be viewed with some degree of caution.

In any case, it is evident from these data that there was no 10 % loss of ^223^Ra from either of the solutions during these measurements.

#### 3.2.2 Experiment 3

The sole purpose of this experiment was to test the stability of the diluent composition that was used in the 2008 standardization to determine if the presence of Sr^+2^ ions or large dilution factors could induce loss of ^223^Ra activity during transfers. Therefore, the only measurements of interest were the ratios between the acid ampoules, which were observed to be stable based on the data from Experiment 2, and the citrate ampoules prepared using the “old” diluent composition.

The activity ratios for ampoules representing the different stages of the dilution scheme, as measured by the AutoIC, γ-spectrometry, and mass determinations, are presented in Table 2. With the exception of the C-A3:A-A3 ratio, all of the values agree to within their respective uncertainties. In the case of the C-A3:A-A2 ratio, the agreement between the radiometric and gravimetric values is about 1.3 %. While this is significant because we would have expected the best agreement to be between ampoules of the first level dilution, it does not provide any evidence (to within the limits of the uncertainty) to support the hypothesis of ^223^Ra loss during any of the transfers.

### 3. 3 Activity Standardization Measurements

#### 3.3.1 LTAC

As an attempt to linearize the extrapolation of the relationship between LTAC count rate and detection inefficiency, an *effective* inefficiency, *Y*, was determined empirically to be

$$Y=0.5Y_{1}+1.0Y_{2}+0.1Y_{3}.$$ (1)

**Fig 4:**
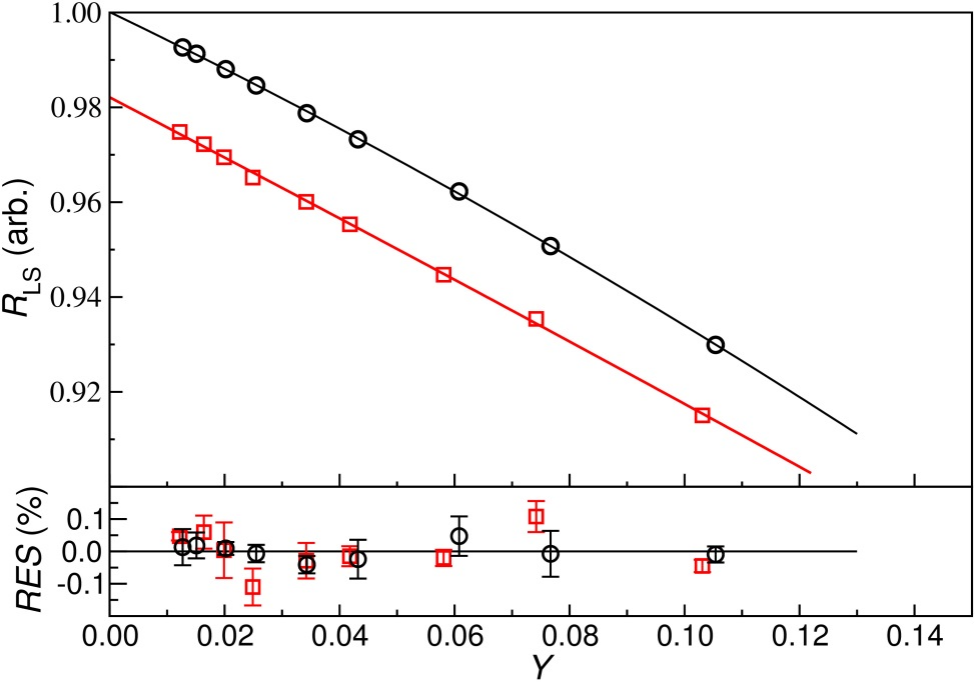
Extrapolation (top) of LTAC LS rate, and percent residuals (bottom) vs. effective inefficiency, *Y*, for acid (circles) and citrate (squares) sources. Uncertainty bars are standard deviations of the mean repeated measurements over 20 days. No residual trend is evident in the acid data, but a small trend may be present in the citrate data.

Here *Y_i_* are the anti-coincident to total γ-ray count-rate ratios for each of the three gates used. Since the highest-energy beta decays from ^211^Pb (*E_β_* = 1367 keV) and ^207^Tl (*E_β_* = 1418 keV) did not coincide with γ-rays, the extrapolation is expected have a small non-linear component. A quadratic extrapolation from 0.012 < *Y* < 0.11 and a linear extrapolation from 0.012 < *Y* < 0.044 both gave adequate fits, which produced intercepts that differed by 0.05 %. The quadratic fit and residuals for the average of 6 measurements on 4 acid-series sources are shown in Fig. 4. The absence of a trend in the residuals indicates that the fit was adequate. The citrate-based data were similar, with a small trend (0.1 %) possibly evident in the residuals. The between-measurement standard deviation of the distribution for the acid sources was 0.06 % and 0.19 % for the citrate. Again, this seemed to indicate larger variability in the citrate data. Neither showed a trend with time during the 20 days of measurements. No significant source-to-source difference was seen in either set of sources.

**Fig. 5:**
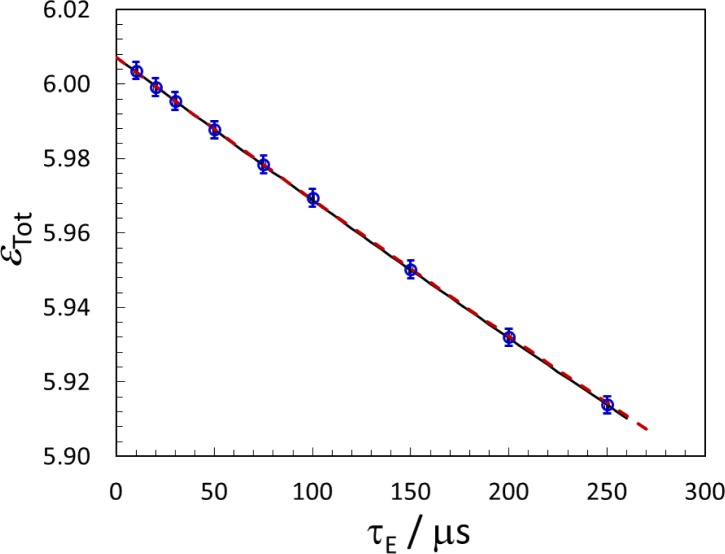
Measurements of LTAC LS intercept efficiency (ε_Tot_) for a single data set analyzed using various extending dead times (τ*_E_*). The uncertainties are from the efficiency extrapolations, so are strongly correlated. The black solid line is a quadratic fit, which was used to normalize the data to an intercept of 6.007 (see text). The red dashed line is calculated from Eq. (2) with no free parameters.

The LS extrapolation intercept, *R*_0_, corresponds to 100 % LS efficiency for both *α*- and *β*-decay. From the Bateman equations, the total chain efficiency would then be ε_Tot_ = 6.0072(1). However, since the ^215^Po half life is only *T*_Po_ = 1.781(4) ms [13], some of its decays will be lost in the extending-dead-time, *τ_E_*, following the decay of its parent, ^219^Rn. For τ_E_ = 50.5 μs, the ^215^Po non-loss is calculated to be 0.9805, and ε_Tot_ = 5.9877. To check this calculation, a single LTAC list-mode data set, comprising 1.4 × 10^4^ s of counting data, was analyzed for various τ_E_ values. The resultant *R*_0_ values were fit with a quadratic function and are shown in Fig. 5 as total efficiency, ε_Tot_. This efficiency was also calculated from the Bateman equations, modified for the dead-time correction,

ϵTot=(5.0072+2−τETPo). (2)

The calculated function (Fig. 5, dashed line) is in good agreement with the data. The value of ε_Tot_ at τ_E_ = 50.5 μs is 5.9877 and 5.9876 from the calculation and quadratic fit, respectively.

Another concern with this decay chain is the presence of ^219^Rn in the LS source. The LTAC method relies on the assumption that as the lower level discriminator of the LS detector is reduced toward zero, the efficiencies for all nuclides in the chain tend toward 100 %. However, if some of the ^219^Rn escapes into the air space in the source vial, then the efficiency for the alpha particles decaying from those gaseous ^219^Rn nuclides will be governed by geometry rather than the discriminator setting. If that situation exists, then the extrapolation intercept would not necessarily be the expected total activity.

The amount of ^219^Rn in the gas phase is expected to be small, due to the known affinity of organic solvents for Rn [20] and the short (4 s) ^219^Rn half-life. To estimate the concentration of ^223^Ra in the gas phase, the LS alpha efficiency for the lowest threshold was monitored using NaI gates G1 and G2. Correcting this value for the presence of γ-rays from beta emitters and aqueous alpha emitters, the ^219^Rn efficiency is measured to be 0.998(3). Furthermore, if Rn is leaving the solution, then the intercept should depend on the linear combination of gates. In particular, if only G3 is used, then a 10 % gas fraction would lead to a 1.2 % change in the intercept. In the present data, if only G3 is used, the intercept changes by only 0.07 %. Lastly, two different sized LS hemisphere vials were used, one with approximately double the air space as the other. The difference in measured activity in the two sizes was only 0.02(10) %. From the combination of all these checks, we conclude that no correction is needed for Rn in the gas phase, confirming our earlier findings during the 2008 experiments [1].

The LTAC activity measurement results are as follows. The activity concentration of solution A-D2 was found to be 4.143 × 10^3^ Bq·g^−1^ and that of C-D2 was found to be 4.218 × 10^3^ Bq·g^−1^. Applying the gravimetric dilution factor of 193.73 for the acid series, the activity concentration of the master solution was calculated to be 8.028(17) × 10^5^ Bq·g^−1^. Applying the gravimetric dilution factor of 186.90 for the citrate series, the activity concentration of the master solution was found to be 7.884(17) × 10^5^ Bq·g^−1^, which is 1.8 (3) % lower than the value obtained for the acid series. The observed difference between the activity concentrations of the two solutions is possibly due to losses of activity in the citrate solutions during source preparation, although no losses were evident based on the gravimetric dilution factors. The uncertainty analysis for the determination of the activity concentration of the acid solution is shown in Table 3.

One of the hemispheres that were prepared from the acid series was counted again in the LTAC system 8 months after preparation in order to look for possible long-lived impurities. None were observed to within an LS impurity limit of about 0.004 % of the ^223^Ra activity at the reference time. In the alpha counting window, the limit was about 0.0007 %, and in the NaI gamma window the limit was about 0.05 %.

#### 3.3.2 TDCR

Activity concentration values for each individual TDCR measurement were calculated using a second-order polynomial fit to data provided by Kossert [21] obtained by using the MICELLE2 code [22]. The data consisted of logical-sum-of-doubles efficiencies calculated as a function of the triples-to-doubles coincidence (T/D) ratio, taking into account the equilibrium activity ratios for all the decay chain members. A correction of 1.9 % for counting losses due to the short half-life of ^219^Po and the 50 μs imposed extending dead time of the TDCR system was also made to the ^219^Po detection efficiency.

A preliminary analysis of the data showed a clear difference in the massic activities for the citrate and acid solutions that was not accounted for by the respective gravimetric dilution factors back to the master solution. The data from each solution series were therefore analyzed as two distinct sets.

Analysis of variance (ANOVA) on the data sets from each of the solutions indicated that it was appropriate to consider all the counting data within each solution series as a single group. This includes the overnight counting data for one of the acid series sources. As a result, an activity concentration of 4.128 × 10^3^ Bq·g^−1^ was determined from the average of 159 measurements made on solution A-D2, which leads to an activity concentration of 7.998(15) × 10^5^ Bq·g^−1^ for the master solution when the appropriate dilution factors are applied. Using the 45 measurements for the citrate solution, an average value of 4.186 × 10^3^ Bq·g^−1^ for solution C-D2 was obtained, leading to a value of 7.823(15) × 10^5^ Bq·g^−1^ for the activity of the master solution. In both cases, the uncertainties cited are combined standard uncertainties calculated from the components presented in Table 4.

The difference between the LTAC and TDCR results for the acid solution was 0.38 % and was within the respective experimental standard uncertainties (*p* = 0.096 from Student t-distribution for equal means). For the citrate solution, the results showed a difference of 0.78 %. In this case, the results were not in agreement (*p* = 0.005 from Student t-distribution for equal means). As discussed above, this was taken to be another indication of the instability of the citrate solution.

It is customary in our laboratory to adopt the result of the “best” method as the activity of the solution being measured regardless of the number of techniques used. The two primary techniques (LTAC and TDCR) applied in this study both gave results that were in agreement with each other, with very similar combined standard uncertainties. We have adopted the LTAC values for the acid series as the reference activity that will be used to calculate all new calibration factors for IC “A” and the VIC since LTAC utilizes a much simpler and more tractable model for determining the activity.

**Fig. 6:**
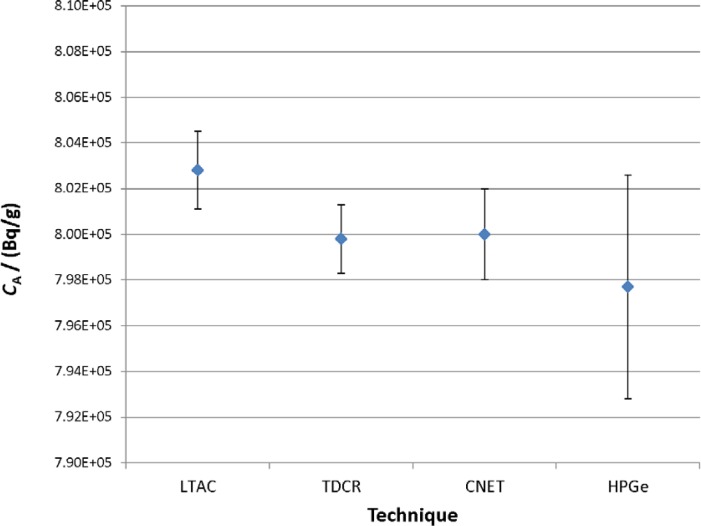
Summary of activity concentration results for the acid series master solution in Experiment 2 as measured by the four techniques used in this study. The uncertainty bars correspond to a single uncertainty (*k*=1) interval and are the combined standard uncertainties on the activity concentrations, calculated as described in the text.

A plot of the activity concentrations for the acid series master solution, as determined from the two primary methods and the two confirmatory measurement techniques used in this study, is given in Fig. 6.

### 3.4 Confirmatory Activity Measurements

#### 3.4.1 CIEMAT-NIST efficiency tracing

At the time that the experimental plan was devised for Experiment 2, it was decided that CNET results would be used solely as a confirmation of the LTAC and TDCR measurements. Since the LS detection efficiency for ^223^Ra is so high (about 600 %) and does not vary much over even relatively large quenching ranges, no ^3^H sources were prepared to do a direct efficiency tracing. Instead, typical ^3^H efficiencies obtained in our laboratory for similarly prepared LS sources counted on the identical instruments with similar quench indicating parameters were used to provide an estimate of the ^223^Ra efficiency using the ^3^H to ^223^Ra efficiency relationship established in Cessna and Zimmerman [1]. These efficiencies were then used to calculate the activity concentrations of the LS sources for the citrate and acid solutions C-D2 and A-D2, respectively. The average efficiency used in the calculations was 599 %. Efficiencies were compared with data provided by Kossert [21] indicating that the NIST calculated total efficiencies were approximately 0.5 % higher than the PTB calculated efficiencies for a ^3^H efficiency of 31 %. This corresponds to a 0.1 % difference in recovered activities. The majority of this difference is most likely attributed to the calculated losses for the short-lived daughter ^215^Po incorporated in the PTB efficiencies. The NIST efficiency does not incorporate a correction, rather it places a limit on the loss based on the 2008 measurements [1].

The activity concentrations recovered for citrate and acid solutions C-D2 and A-D2 are 4.03 × 10^3^ Bq·g^−1^ and 4.28 × 10^3^ Bq·g^−1^, respectively. Incorporating the dilution factors gave values of 7.81(4) × 10^5^ Bq·g^−1^ and 8.00(2) × 10^5^ Bq·g^−1^ for the master solution based on the citrate and acid dilutions, respectively. As with the other methods the citrate activity is seen to be 2.3 % low relative to the acid solution. The uncertainties were dominated by components associated with the counting of the LS sources. An ANOVA of all data sets did not support combination of results from individual sources or data acquired on different LS counters. Therefore, components are given for between-cycle, between-source, and between-counter evaluated uncertainties. The latter was the largest component for the citrate based dilution. A visual analysis indicated a slight instability with recovered activities dropping by approximately 0.75 % percent over 5.5 days. Details of the uncertainty components are given in Table 5.

#### 3.4.2 γ-ray spectrometry

The total activity per source for each gamma-ray line, at the same reference time used for all the other techniques, for each of the ^223^Ra sources was determined using Eq. (3).

$$A=\frac{N(E)}{T\;P_{\gamma }(E)\;\epsilon (E)}\prod_{i}C_{i}$$ (3)

where *A* is the source activity, *P_γ_*(*E*) is the emission probability for each gamma-ray line of energy *E*, *N* is the net area under the peak for each gamma-ray spectral line, *T* is the live time of the measurement, ϵ(*E*) is the full-energy-peak efficiency for each gamma-ray energy and *C_i_* are the correction factors applied to the measurements. For these measurements, three correction factors were applied to account for: random pile-up counting, differences in the source mass (that translates into a solution height correction) and source decay to the reference time. The time available to perform the measurements was short due to the relatively short ^223^Ra half-life, thus random pile-up corrections were necessary. These corrections varied between 0.01 % and 4 %, depending on which HPGe detector and source-to-detector distance were used for the measurements. The solution height corrections varied between 0.1 % and 0.4 %. At the various measurement distances the full-energy-peak efficiencies varied between 1 × 10^−4^ and 4 × 10^−5^.

The tabulated emission probabilities obtained from DDEP [13] and the National Nuclear Data Center (NNDC) Evaluated Nuclear Structure Data File [23] for the main gamma-rays in the ^223^Ra decay chain were initially used for the source activity determinations. The initial calculated values were found to have an unacceptably high level of variability from the use of those data. Subsequently, emission probabilities measured by NPL [19] were used to calculate the source activities. The emission probabilities used for these measurements are listed in Table 6.

The average activity was computed using the 56 values obtained from the eight measurements of each source and the seven main gamma-ray lines. These values were used to create a matrix with 56 rows (1 row per measurement per line) and 16 columns (that list the value for each variable in Eq. (3) plus the source mass and their associated uncertainties). This matrix was used to compute the correlation coefficients using the procedure defined in the Guide to Expression of Uncertainty in Measurement [24] using the RStudio software (RStudio, Boston, MA), in order to obtain the uncertainties.

From the acid series, the average activity concentration of the master solution was found to be 7.98(5) × 10^5^ Bq·g^−1^, whereas for the citrate master solution, the average activity concentration was found to be 7.97(7) × 10^5^ Bq·g^−1^. These values are the average of the activity concentrations obtained from X-A1, X-A2, and X-A3, taking into account the gravimetric dilution factors to the ^223^Ra master. The uncertainties take into account the typical uncertainty on the measurement of an individual ampoule (0.6 % for acid series, 0.8 % for the citrate) and the standard deviations on the three ampoules in each set (0.17 % for the acid, 0.23 % for the citrate).

An analysis for photon-emitting impurities, performed on ampoule A-A1 from Experiment 2, showed no impurities. The minimum detectable activity for the impurities, per Becquerel of ^223^Ra at the reference time for photons with energy *E*, was measured to be

$$\begin{array}{l} 40\;\mathrm{keV}<E<780\;\mathrm{keV}\;6.7\times 10^{-3}\;\text{to}\;1.2\times 10^{-3}\;\text{photons per second}\\ 790\;\mathrm{keV}<E<2000\;\mathrm{keV}\;2.4\times 10^{-3}\;\text{to}\;8\times 10^{-5}\;\text{photons per second}, \end{array}$$

assuming that the impurity photon energy is more than 5 keV from any photon emitted in the ^223^Ra decay chain.

### 3.5 Comparison of Acid and Citrate Solution Activities

**Fig 7:**
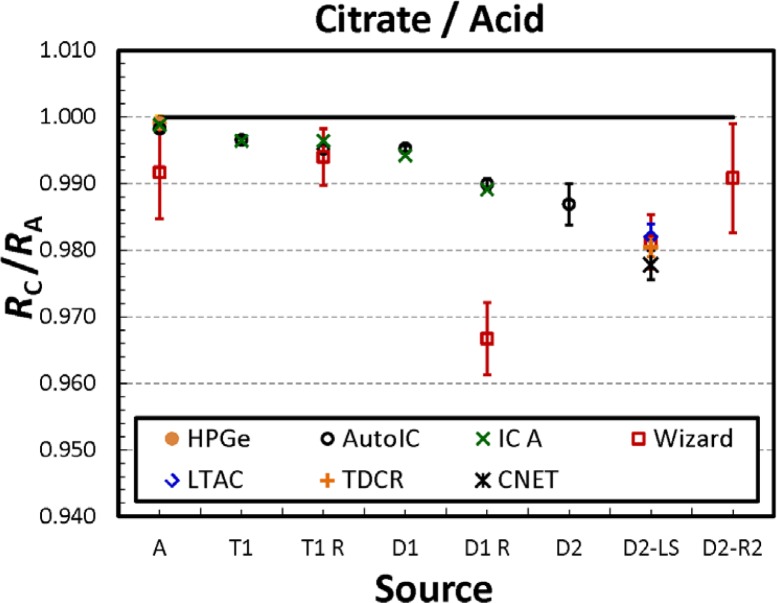
Ratios R_C_/R_A_ for relative measurement response determined for the citrate and acid solutions in Experiment 2. The values are calculated as the ratio of the decay- and background-corrected rates (or ionization current, in the case of ionization chamber measurements) for the citrate and acid sources that have been divided by their respective source masses and the product of the respective dilution factors leading to the master solution. The uncertainty bars correspond to a single uncertainty interval calculated only from the standard deviations of the mean for the respective measurements.

Comparing the relative measurement responses of the citrate solution sources to the corresponding acid solution source for the same dilution level in Experiment 2 using all the available techniques, we observe a decrease in the apparent citrate solution activity as a function of the dilution level. This is shown in Fig. 7. The ratios presented in the plot are calculated as

$$\frac{R_{C}}{R_{A}}=\frac{\left(R_{x,C}-R_{x,B}\right)/\left(m_{C}\prod D_{C,i}\right)}{\left(R_{x,A}-R_{x,B}\right)/\left(m_{A}\prod D_{A,i}\right)}$$ (4)

where *R_x,_*_C_, *R_x,_*_A_, and *R_x,_*_B_ are the decay-corrected responses (ionization current or counting rates) of the respective citrate, acid, or background source for each dilution level; *m*_C_, and *m*_A_ are the masses of the respective citrate and acid source; and Π*D*_C,_*_i_* and Π*D*_A,_*_I_* are the products of the dilution factors from that particular dilution level for the respective citrate and acid dilution chains leading to the master solution.

From these data, a nearly 2 % drop in activity can be observed in the citrate-prepared solution sources relative to the acid series for all of the techniques used over the entire dilution chain. Any analogous trend in the Wallac Wizard data is obscured by scatter. Note that the uncertainty bars in Fig. 7 represent only the standard deviations on repeated measurements. Other uncertainty components are not included.

The downward trend observed in Fig. 7 is a subtle effect between dilution steps and is most noticeable when examined as a cumulative difference between the first and last steps.

There is no evidence for this effect in Experiment 3, however. Although the composition of the citrate solution was slightly different (by the addition of trace amounts of Sr^+2^ ions) from the one used in Experiment 2, it was expected to be identical to the one used in the 2008 standardization. Solution stability losses are rarely reproducible, thus the lack of consistency in the magnitude of the observed losses from the citrate solution (Tables 1 and 2) is perhaps not surprising.

This demonstrates the need to monitor the activity concentration radiometrically whenever transfers or dilutions of the citrate solution are made. Although the final dilution step in these present experiments could only be confirmed to about 2 % due to poor counting statistics, if this approach had been taken in 2008, it is quite probable that the 10 % discrepancy in the 2008 results would have been identified.

### 3.6 Comparison to 2008 Secondary Standard

From the data taken in the IC “A” for ampoules A-A1 and C-A1 and using the calibration coefficient determined in the 2008 standardization and the respective gravimetric dilution factors, we obtained activity concentrations of 7.25(3) × 10^5^ Bq·g^−1^ and 7.26(3) × 10^5^ Bq·g^−1^ for the acid and citrate solutions, respectively. This corresponds to respective differences of −9.5 % and −9.6 % for the two solutions relative to the LTAC acid series measurements.

Using the calibration coefficient for the Vinten 671 chamber given in Bergeron, *et al.*, we obtained an average activity concentration of 7.28(6) × 10^5^ Bq·g^−1^ for the acid solution. It should be noted, however, that a different source holder was used for the determination of the 2008 K_VIC_ value than was used in this study. Comparative measurements in the two geometries indicated that this causes a +0.5 % effect on the calculated activity, which would give a value of 7.24(6) × 10^5^ Bq·g^−1^ if the measurement had been made in the holder used in the present studies. This is in agreement with the results of IC “A” and indicates a difference of 9.3 % relative to the LTAC measurements.

## 4. Conclusion

As a result of a discrepancy involving our published secondary standards for ^223^Ra brought to our attention by colleagues at the National Physical Laboratory, we have undertaken a thorough review of our 2008 work and performed two large studies to re-standardize this radionuclide that will hopefully provide an explanation for the source of the discrepancy. The new studies confirmed a difference of −9.5 % between the previous secondary standard and the new primary standard, and this new higher value is in agreement with the new standard published by NPL. This new primary standard is based on measurements made with methods that are taken to be more robust than those available in the original 2008 experiments.

The new studies revealed the presence of instability in the citrate solution when dilutions or transfers are performed that resulted in a loss of about 2 % of the ^223^Ra that was not accounted for by the gravimetric dilution factors. This was confounded by the fact that this effect was not reproduced in a second experiment. Although this was not of the magnitude of the discrepancy between the present standard and the one developed in 2008, these data taken together suggest that care must be taken to verify the activities of the citrate solution whenever transfers or dilutions of these solutions are made.

## Figures and Tables

**Table 1a:** Ratios of measured gravimetric dilution factors, along with ratios of mass-normalized count rates (for Wallac Wizard), ionization currents (for Vinten671, IC “A”, and AutoIC), or ^223^Ra activities (γ-spectrometry) for ^223^Ra acid ampoule sources prepared in Experiment 2. The uncertainties quoted are standard uncertainties calculated from the quadratic addition of the standard deviations (or standard deviations of the mean for the Vinten 671 measurements) on the appropriate values. The standard uncertainties on the gravimetric mass ratios are the quadratic combinations of the standard uncertainties arising from the agreement between the dilution factor values calculated using both added and contained masses.

Ratio	Mass	Vinten 671	IC “A”	AutoIC	γ-spec	Wallac Wizard
**A-A1:Acid Master**	1					–
**A-A2:Acid Master**	1	1.000(7)	1.0000(2)	1.000(1)	1.000(1)	–
**A-A3:Acid Master**	1					–

**A-A1:A-T1**	1	1.001(1)	1.0006(3)	1.0008(3)	–	–
**A-T1:A-T1 R**	1.314961(7)	1.320(3)	1.3147(10)	1.3152(5)	–	–

**A-T1:A-D1**	3.808053(88)	3.8(2)	3.806(4)	3.803(2)	–	–
**A-D1:A-D1 R**	1.07312(3)	1.08(1)	1.073(1)	1.075(2)	–	–
**A-T1 R :A-D1 R**	3.107689(83)	3.12(3)	3.107(3)	3.108 (2)	3.10(6)	3.121(7)

**A-D1:A-D2**	16.0905(16)	–	–	16.07(4)	–	–
**A-D1 R:A-D2 R**	16.673(2)	–	–	–	16.4(7)	16.9(3)
**A-D2:A-D2 R**	1.111914(9)	–	–	–	–	1.11(2)

**Table 1b:** Ratios of measured gravimetric dilution factors, along with ratios of mass-normalized count rates (for Wallac Wizard), ionization currents (for Vinten671, IC “A”, and AutoIC), or ^223^Ra activities (γ-spectrometry) for ^223^Ra citrate ampoule sources prepared in Experiment 2. The uncertainties quoted are standard uncertainties calculated from the quadratic addition of the standard deviations (or standard deviations of the mean for the Vinten 671 measurements) on the appropriate values. The standard uncertainties on the gravimetric mass ratios are the quadratic combinations of the standard uncertainties arising from the agreement between the dilution factor values calculated using both added and contained masses.

Ratio	Mass	Vinten	IC “A”	AutoIC	γ-spec	Wallac Wizard
**C-A1:Citrate Master**	1					–
**C-A2:Citrate Master**	1	1.0000(9)	1.0000(2)	1.0000(2)	1.000(1)	–
**C-A3:Citrate Master**	1					–

**C-A1:C-T1**	1	1.006(2	1.0017(3)	1.0009(3)	–	–
**C-T1:C-T1 R**	1.309083(51)	1.306(3)	1.310(1)	1.3121(7)	–	–

**C-T1:C-D1**	3.97209(48)	4.02(2)	3.979(4)	3.975(2)	–	–
**C-D1:C-D1 R**	1.07216(14)	1.08(1)	1.078(1)	1.0799(7)	–	–
**C-T1 R:C-D1 R**	3.2532(6)	3.33(3)	3.278(3)	3.272(2)	3.24(7)	3.2(1)

**C-D1:C-D2**	15.5378(95)	–	–	15.64(3)	–	–
**C-D1 R:C-D2 R**	16.62(1)	–	–	–	16.7(4)	16.8(6)
**C-D2:C-D2 R**	1.147531(72)	–	–	–	–	1.15(2)

**Table 2:** Ratios of measured gravimetric dilution factors, along with ratios of mass-normalized ^223^Ra activities for ^223^Ra citrate and acid ampoule sources for the sources prepared in Experiment 3. The uncertainties quoted are standard uncertainties calculated from the combined standard uncertainties on the measurements used for the ratios. Details of the uncertainty assessments for the individual techniques are given in the text. The standard uncertainties on the gravimetric mass ratios are the quadratic combinations of the standard uncertainties arising from the agreement between the dilution factor values calculated using both added and contained masses.

Ratio	Mass	AutoIC	γ-spec
**A-A3:C-A3**	0.9819(2)	0.9945(5)	0.991(3)
**A-D2:C-D2**	1.381(2)	1.379(4)	1.373(6)
**A-D1 R:C-D1 R**	0.97044(2)	0.9699(3)	0.972(3)
**A-D1 R:A-D2**	38.61(4)	38.52(6)	38.5(2)
**C-D1 R:C-D2**	54.92(7)	54.75(13)	55.0(4)
**A-D2-T:A-D2**	0.9918(1)	1.001(9)	1.00(2)
**C-D2-T:C-D2**	0.9930(1)	0.995(14)	1.00(2)

**Table 3:** Uncertainty analysis for the massic activity of the master solution using the acid-series LTAC data.

Name	Description	Type	*u_i_* (%)
**Measurement Variability**	Standard deviation of the distribution for 6 measurements using 4 sources, and 4 background runs using 2 blanks, over 20 days.	A	0.06
**Background**	Standard uncertainty on *C*_A_ due to variability in background. Partially accounted for by Measurement Variability. Taken to be 1/2 the difference between nominal activity and that using the same background run for all sources.	A	0.03
**Gravimetric Links**	Standard uncertainty on *C*_A_ due to uncertainty in both the dilution factors (DF) and LS source masses. Assume 0.05% correlated uncertainty in LS source masses (50 mg) and 0.01 % uncertainty in each DF (from dispensed-contained mass agreements).	B	0.05
**Solution stability**	No losses seen. Limit on loss of activity during source preparation. Taken as 1/2 the difference between the gravimetric dilution factors and those recovered from relative activity measurements.	A	0.10
**Extrapolation**	Standard uncertainty on *C*_A_ due to extrapolation of the relationship between counting rate and detection inefficiency. Typical (median) uncertainty in the intercept of a quadratic extrapolation, added in quadrature with the average difference between linear and quadratic extrapolations (0.05 %).	A	0.10
**Live-Time**	Standard uncertainty on *C*_A_ due to uncertainty in the live-time counting (minimum 90 % live) from limit of previous tests.	B	0.1
**Veto losses of Po-215**	Standard uncertainty on *C*_A_ due to the uncertainty on the 0.3 % correction to the LS intercept due to Po-215 pulses being vetoed by extending-dead time. Taken to be the difference between calculated and extrapolated correction factor.	B	0.002
**Radioactive Equilibrium**	Standard uncertainty on *C*_A_ due to calculation of equilibrium activity ratios for the members of the Ra-223 decay chain. From DDEP evaluation of half-lives.	B	0.002
**Decay correction**	Standard uncertainty on *C*_A_ due to 0.26 % uncertainty in the ^223^Ra halflife.	B	negligible
**Impurities**	No impurities found. Based on upper limit of ^227^Th from HPGe spectrometry.	B	0.09
		***u****_c_*	**0.21**

**Table 4:** Evaluated uncertainty components for the measurement of the ^223^Ra master solution by the Triple-to-Double Coincidence Ratio (TDCR) method.

Name	Description	Type	*u_i_* (%), citrate	*u_i_* (%), acid
**Measurement repeatability**	Standard deviation on average activity value from measurement of 3 sources, measured with a minimum of 3 gray filters on between 1 and 3 occasions. Between 20 and 108 measurements for each source for the acid series, between 9 and 189 measurements on each source for the citrate.	A	0.017	0.051
**Background variability**	Standard uncertainty on *C*_A_ due to uncertainty in triple and double background counting rates. One background cocktail for each of the two diluents were counted three times at each of two gray filters (maximum and minimum darkness). Typical standard deviation on counting rates was 1.2 %.	A	0.0050	0.0010
**Decay correction**	Standard uncertainty on *C*_A_ due to 0.26 % uncertainty in the ^223^Ra half-life. Decay corrections were carried out over times ranging from 0 d to 7 d from the reference date	B	0.11	0.11
**Mass determinations**	Standard uncertainty on *C*_A_ due to LS cocktail mass measurements.	B	0.05	0.05
**Dilution factor**	Standard uncertainty on *C*_A_ due to uncertainty in dilution factor from LS sources to master solution.	B	0.045	0.011
**Counting statistics**	Standard uncertainty on *C*_A_ due to “Poisson counting error”.	A	0.10	0.10
**TDCR/efficiency determination**	Standard uncertainty on *C*_A_ due to uncertainty in efficiency estimate and in measurement of experimental TDCR.	B	0.10	0.10
**TDCR dead time**	Standard uncertainty on *C*_A_ due to uncertainty on calculated doubles efficiency due to uncertainty in the extending dead time.	B	0.05	0.05
		***u****_c_*	**0.19**	**0.19**

**Table 5:** Evaluated uncertainty components for the measurement of the ^223^Ra master solution by the CIEMAT/NIST ^3^H efficiency tracing (CNET) method.

Name	Description	Type	*u_i_* (%), citrate	*u_i_* (%), acid
**Measurement repeatability**	Within-source measurement variability. Standard deviation on average *C*_A_ value from *n*=10 measurements of each source.	A	0.15	0.12
**Measurement reproducibility**	Between-source measurement variability. Standard deviation on average *C*_A_ value from *n*= 5 sources.	A	0.20	0.13
**LS counter dependence**	Standard deviation on average *C*_A_ value from3 commercial LS counters.	A	0.33	0.05
**Background variability**	Standard uncertainty on *C*_A_ due to uncertainty in background counting rates. Typical standard deviation on counting rates was between 2.5 % and 6.0 %.	B	0.002	0.003
**Decay correction**	Standard uncertainty on *C*_A_ due to 0.26 % uncertainty in the ^223^Ra half-life. Decay corrections were carried out over times ranging from 3 d to 7 d from the reference date	B	0.07	0.07
**Mass determinations**	Estimated standard uncertainty on *C*_A_ due to LS cocktail mass measurements.	B	0.05	0.05
**Dilution factor**	Standard uncertainty on *C*_A_ due to uncertainty in dilution factor from LS sources to master solution.	B	0.045	0.011
**Livetime**	Estimated standard uncertainty on *C*_A_ due to uncertainty in determination of LS counter livetime.	B	0.05	0.05
**CNET efficiency determination**	Standard uncertainty on *C*_A_ due to uncertainty in efficiency estimate. Taken as the 0.28% range of estimated efficiencies.	B	0. 05	0.05
**Activity of ^3^H standard**	Estimated standard uncertainty on *C*_A_ due to 0.36% uncertainty in ^3^H standard activity.	B	0.05	0.05
**Branching ratios**	Estimated uncertainty on *C*_A_ due to uncertainty in decay branching ratios	B	0.18	0.18
		***u****_c_*	**0.47**	**0.28**

**Table 6:** Emission probabilities for ^223^Ra decay chain used in the determination of ^223^Ra solution source activity using HPGe gamma-ray spectrometry [19].

Radionuclide	Energy (keV)	Emission Probability per Disintegration
**^223^Ra**	122.319	0.01311 ± 0.00019
	144.27	0.0349 ± 0.0005
	154.208	0.0604 ± 0.0009
	269.463	0.1342 ± 0.0016
	323.871	0.0369 ± 0.0005
	338.282	0.0262 ± 0.0004
	445.033	0.01225 ± 0.00016

## Footnotes

1 Certain commercial equipment, instruments, or materials are identified in this paper to foster understanding. Such identification does not imply recommendation by the National Institute of Standards and Technology, nor does it imply that the materials or equipment identified are necessarily the best available for the purpose.
